# Systemic Barriers and Emotional Complexities in the Transition to Adulthood: The Lived Experience of Parents of Individuals With Intellectual Disabilities in Italy

**DOI:** 10.1111/jar.70294

**Published:** 2026-08-14

**Authors:** Mariachiara Feresin, Elena Bortolotti

**Affiliations:** ^1^ Department of Human Studies University of Trieste Trieste Italy

**Keywords:** challenges, intellectual disabilities, life project, parental experiences, transition to adulthood

## Abstract

**Background:**

The transition to adulthood for individuals with intellectual disabilities (ID) is a complex process marked by significant challenges for families.

**Method:**

Using semi‐structured interviews and thematic analysis with 17 parents of individuals with ID, this qualitative research examined transition obstacles, support systems, and emotional responses impacting family reorganization and life projects (LP) definition.

**Results:**

Parents reported difficulties in planning and implementing LP due to standardized approaches, a lack of structured employment pathways, and challenges navigating fragmented services. Emotionally, parents exhibited resilience, pride, and hope, alongside anxiety, frustration, and powerlessness regarding their children's future and the adequacy of support.

**Conclusions:**

The findings emphasize the need for holistic, family‐centered support systems addressing practical and emotional needs. The LP framework is promising, but successful implementation requires bridging systemic gaps, enhancing service coordination, and empowering parents to improve outcomes for individuals with ID.

## Lay Summary

- The study revealed significant systemic challenges faced by parents of individuals with ID during the transition to adulthood, including inadequate life project planning, limited employment opportunities, and difficulty navigating fragmented support services.
- Parents experience a complex range of emotions, demonstrating resilience, pride, and hope while also grappling with anxiety, frustration, and a sense of powerlessness in advocating for their children.
- The findings highlight the critical need for more tailored, coordinated, and accessible support services to ease the burden on families and effectively implement frameworks like the LP, which aims to promote autonomy and inclusion for individuals with ID.
- For the research community, this study underscores the need for future research to evaluate the effectiveness of current policies and integrate the perspectives of individuals with ID themselves.

## 1 Introduction

Parenthood in the context of intellectual disability (ID), particularly during the transition to adulthood, represents a complex and non‐linear process shaped by the intersection of family dynamics, caregiver well‐being, service systems, and individual developmental trajectories. Within the literature, developmental transitions are frequently conceptualized as non‐normative critical events that challenge family equilibrium, elevate parental stress, and require coordinated, multi‐sectoral support (Kanthasamy et al. 2024; Franklin et al. 2019; Mkabile and Swartz 2020; Brown et al. 2019). The transition from adolescence to adulthood is especially salient for families of young people with ID, as it introduces heightened uncertainty regarding autonomy, independent living, employment opportunities, and long‐term societal participation.

The birth of a child with ID is often described as a normative life event that becomes non‐normative when atypical development emerges, increasing the risk of family maladjustment. This framing extends into the transition to adulthood, which constitutes a further critical juncture with potential repercussions for family stability and well‐being. During this phase, families must navigate a shift from child‐centered to adult‐oriented service systems, frequently encountering fragmented planning, insufficient coordination, and services that are poorly aligned with individual and family needs (Franklin et al. 2019; Vogan et al. 2014; Garrels and Sigstad 2023; Brown et al. 2019). Such systemic discontinuities amplify caregiver stress and reinforce perceptions of institutional gaps.

Within this context, family resilience emerges as a central protective and adaptive resource. Resilience is commonly defined as the family's capacity to cope with and rebound from prolonged critical events through adaptive strategies and relational processes. Drawing on Walsh's framework, resilience is shaped by the duration and phase of the critical event, the family life cycle stage, and the availability of internal and external supports (Kanthasamy et al. 2024; Franklin et al. 2019). In the transition‐to‐adulthood literature, resilience manifests concretely in families' ability to access services, engage in advocacy, support the development of self‐determination in their young adult, and maintain a future‐oriented and hopeful perspective. Engagement with professionals, proactive information‐seeking, and collaborative problem‐solving are consistently associated with more positive transition outcomes, underscoring resilience as a relational and systemic process rather than merely an individual trait (Wilt et al. 2020; Leonard et al. 2016; Codd and Hewitt 2020; Mkabile et al. 2021).

At the same time, caregiver burden and psychological distress are widely documented in families of children with disabilities and tend to intensify during transitional periods. Elevated levels of stress and depressive symptoms are reported when families experience uncertainty, insufficient planning, and abrupt shifts from paediatric to adult services (Kanthasamy et al. 2024; Franklin et al. 2019; Vogan et al. 2014; Mkabile and Swartz 2020; Garrels and Sigstad 2023). Importantly, caregiver burden is not uniformly negative. Many parents describe experiences of growth, strengthened coping capacities, and a redefined sense of purpose emerging from supporting their child's path to adulthood, suggesting a nuanced interplay between stress and meaning‐making (Rapanaro et al. 2007). Moreover, caregiver burden is influenced not only by child‐related factors but also by structural determinants, including service inaccessibility, unmet needs, and financial strain, highlighting the centrality of equity and access in transition planning (Vogan et al. 2014; Mkabile and Swartz 2020; Garrels and Sigstad 2023; Mkabile et al. 2021).

System‐level barriers are a recurring theme across international contexts. Health care transition processes are often described as siloed and inefficient, with parents reporting feelings of abandonment and responsibility for independently coordinating complex service networks (Franklin et al. 2019; Brown et al. 2019). Facilitators of smoother transitions include early and explicit planning, inter‐professional coordination, transparent information‐sharing, and sustained collaboration between families and service providers. Conversely, poor communication, delayed planning, and limited information contribute to parental stress and diminished perceived control (Franklin et al. 2019; Brown et al. 2019; Leonard et al. 2016; Codd and Hewitt 2020). Broader barriers—including stigma, fragmented services, limited post‐school opportunities, and restricted social participation for adults with severe ID—further complicate the transition process, particularly in contexts lacking robust policy or community supports (Mkabile and Swartz 2020; Garrels and Sigstad 2023; Mkabile et al. 2021).

Professionals across sectors—including nurses, physicians, educators, employment specialists, and social workers—play a pivotal role in shaping transition experiences. When collaboration is consistent and person‐ and family‐centered, professionals are perceived as instrumental in facilitating smoother pathways to adulthood (Malapela et al. 2020; Brown et al. 2019; Codd and Hewitt 2020). However, qualitative research also highlights tensions between policy discourses emphasizing self‐determination and the lived realities of families whose voices may be marginalized in decision‐making processes (Pilnick et al. 2011; Brown et al. 2019). Negotiating the balance between young adult autonomy and continued parental involvement emerges as a central relational and ethical challenge during transition. In this regard, parental advocacy is increasingly recognized as a key mechanism for securing access to services and mitigating disparities, particularly for youth with ID and co‐occurring conditions (Vogan et al. 2014; Leonard et al. 2016).

Cross‐cutting findings reveal a wide spectrum of parental experiences, ranging from profound anxiety and systemic frustration to personal growth and strengthened family cohesion. This diversity supports a resilience‐oriented framing that moves beyond deficit‐based narratives (Rapanaro et al. 2007; Franklin et al. 2019; Garrels and Sigstad 2023; Leonard et al. 2016). Furthermore, emerging research highlights the compounded barriers faced by immigrant families, socioeconomically disadvantaged groups, and families in low‐ and middle‐income contexts, where language barriers, cultural differences, and financial constraints intensify transition challenges (Nyikach and Hansen 2023; Mkabile et al. 2021; Hronis et al. 2019).

### 1.1 Life Project

The Italian legislative system is evolving to better support individuals with disabilities and their families. The recent Legislative Decree 3 May 2024, n.62, enacted in accordance with Law 227/2021, defines the “Life Project” (LP) as a crucial tool for fostering autonomy, social inclusion, and tailored support for individuals with disabilities. This law signifies a cultural and legislative shift aimed at enhancing the quality of life and promoting full participation in society for people with disabilities.

The LP framework, guided by principles of personalization, participation, multidimensionality, sustainability, and adaptability, is a comprehensive approach to care. This framework moves beyond traditional welfare models by necessitating coordinated and integrated services that foster personalized educational and formative experiences tailored to the individual's future aspirations. Central to the LP are several key tenets: it is rooted in the individual's desires and needs, addresses various life domains including health, education, work, social engagement, housing, and care, ensures long‐term continuity and stability of support, and is continuously monitored and adapted to meet evolving needs.

The Legislative Decree 62/2024 emphasizes key structural components essential for the effective implementation of the LP. The person with a disability is at the heart of the project as the primary beneficiary, while a Multidimensional Assessment Unit, comprised of social workers, physicians, and educators, conducts personalized evaluations to identify specific needs and desires. Families and caregivers are integral to the planning and implementation process, with local authorities, service providers, non‐profit organizations, and healthcare and social assistance programs also contributing to the LP's realization. Adequate financial and human resources, a “Project Budget,” are allocated to support implementation. An Implementation Coordinator ensures seamless coordination among the various services involved, and the LP maintains Portability, remaining valid even if the individual changes residence or context.

Despite these advancements, gaps persist in the provision of adequate and holistic support for families raising individuals with ID.

## 2 Current Study

Building upon these premises, the present study investigates the transition from school to adult life for individuals with ID and their parents, a critical and often complex phase of the life cycle. This transition marks a period of profound family reorganization, where parents face new challenges related to defining their children's LP, activating pathways to independence, and identifying appropriate resources and supports. The post‐school transition, therefore, represents a particularly sensitive period, characterized by uncertainties, expectations, and fears regarding the future, which can significantly influence the emotional well‐being of parents and their ability to support their children along this path.

Within this framework, the overarching aim of the study is to explore the lived experiences of parents of individuals with intellectual disabilities as their children transition to adulthood in Italy. By adopting a qualitative approach, the study seeks to illuminate how parents make sense of this pivotal developmental phase and how they navigate the systemic, relational, and emotional dimensions embedded within it.

More specifically, the study aims to: (a) explore the primary needs and perceived gaps identified by parents during the post‐school transition; (b) examine their expectations, concerns, and representations regarding their children's future; and (c) explore how these perceived needs, expectations, and concerns shape parents' approaches to involvement in the orientation and planning of their children's life projects. Particular attention is devoted to understanding parents' emotional experiences during this transition, with a focus on how they interpret and negotiate the reorganization of family roles and the activation of pathways towards autonomy.

Through giving voice to parents' narratives, this study intends to generate in‐depth insights that may inform the development of more responsive, person‐ and family‐centered support practices, aimed at promoting social inclusion and meaningful adult participation for individuals with ID.

## 3 Methodology

A qualitative research methodology was employed, with semi‐structured interviews conducted with 17 parents to explore their experiences. A semi‐structured format was adopted to ensure consistency across interviews while allowing flexibility to explore emerging themes. Guiding questions invited parents to reflect on areas such as their experiences with planning their child's “life project” (e.g., “How would you describe your experience of planning your child's future after school?”), perceived supports and barriers (e.g., “What kinds of services or resources have been most helpful—or most lacking—during this transition?”), and emotional experiences (e.g., “How have you felt during this phase of transition?”). Follow‐up prompts were used to deepen reflection and clarify meanings, encouraging participants to provide concrete examples from their daily lives. While an effort was made to achieve balanced participation from both mothers and fathers, the final sample included only one father. The disproportionate representation of mothers in research of this nature is a common occurrence, reflecting the persistent gendered division of caregiving responsibilities. The traditional nuclear family model continues to shape caregiving arrangements, resulting in women disproportionately undertaking the majority of both unpaid and paid care work (Chatzidakis et al. 2020; Østerud and Anvik 2024). This study has been conducted in accordance with the ethical standards outlined in the ethical code for research in Pedagogy and it received ethical approval from the Ethical Committee of the University of Trieste.

### 3.1 Data Collection

The key themes explored during the interviews included: a description of the transition experience (school‐to‐post‐school) of their child; the obstacles and facilitators encountered in this process within the educational‐school, community, healthcare, and professional systems; community involvement; social support; and suggestions for improving parent‐professional collaboration to positively support the transition.

We informed presidents of local disability‐related associations about the opportunity for parents of individuals with ID to participate in this research project. The parents themselves, having been informed by the association about the possibility of participating in the study, contacted us by telephone or email. Upon contact, we explained the research project in detail and, if the parent expressed willingness, an interview appointment was scheduled. Prior to each interview, parents were provided with an informed consent form, emphasizing that participation in the research was voluntary. The individual interviews took place from April to July 2024 in an office of the Department of Human Studies at the University of Trieste. The average duration of the interviews was 62 min (range: 32–94 min). Interviews were audio recorded, transcribed, anonymized, and analyzed. In this paper, extracts from participant responses are presented in English, having been translated by the researchers. To ensure anonymity, pseudonyms have been used to identify parents.

### 3.2 Data Analysis

We employed reflexive thematic analysis (Braun and Clarke 2019, 2021) to systematically identify and organize patterns of meaning within the qualitative data, recognizing our active role in constructing themes. This flexible yet methodologically rigorous approach allowed us to explore the nuances of parental experiences during their children's transition to adulthood. We began with in‐depth readings of the transcripts to ensure immersion in the data.

We adopted an inductive coding strategy to ground our analysis in parents' lived experiences and to avoid imposing predefined interpretative frameworks. We then organized initial codes into broader categories aligned with our research aims. Through iterative cycles of comparison and refinement, we developed themes that captured both shared patterns and variations across participants' narratives. Throughout the analytic process, we paid close attention to the emotions expressed by parents and to how these emotional experiences were intertwined with their actions, decision‐making processes, and engagement in their children's transition pathways. In the final phase, we defined, named, and refined themes to construct a coherent narrative addressing our research question and highlighting the interplay between systemic challenges and the emotional dimension of this critical life stage. The data were analysed using NVivo 14 software.

To enhance the credibility and rigour of our findings, we engaged in ongoing peer debriefing throughout the analytic process. We regularly discussed emerging codes, thematic interpretations, and analytic decisions within the research team, critically examining alternative readings of the data and challenging our assumptions. This collaborative process allowed us to refine our interpretations, strengthen the coherence of the thematic structure, and ensure that our findings remained firmly grounded in participants' accounts rather than in our preconceptions.

### 3.3 Participant Characteristics

Sixteen mothers and one father of individuals with ID participated in the study. Thirteen parents were between 50 and 65 years of age, while four were 66 years or older. With regard to employment status, only one mother was unemployed at the time of the study. Six mothers were separated from the father of their child with a disability, and two were widowed. In terms of place of residence, 14 families lived in urban areas, while three resided in rural contexts. Concerning socio‐economic status, the majority of participants described their household situation as middle‐income, with three families reporting lower‐middle economic conditions and three reporting relatively stable upper‐middle economic conditions.

The ages of the individuals with ID ranged from 18 to 35 years. A diagnosis was received within the first year of life for 11 parents' children and after the first year for six. The primary diagnoses were: Down syndrome (2), spina bifida with ID (2), autism (1), ID (7), Prader‐Willi syndrome (1), psychomotor retardation (2), and rare genetic disorder (2). Fifteen individuals with ID required moderate to extensive assistance, while two required minimal assistance. Only two individuals had begun living outside of the family home, both in supported living arrangements with professional assistance tailored to their daily support needs, and none were employed.

### 3.4 Ethical Considerations

This study has been conducted in accordance with the ethical standards outlined in the ethical code for research in Pedagogy and it received ethical approval from the Ethical Committee of the University of Trieste.

All participants were fully informed about the aims and procedures of the study, the voluntary nature of their participation, and their right to withdraw at any time without consequence. Written informed consent was obtained prior to data collection. Particular attention was paid to safeguarding confidentiality and anonymity: all interviews were anonymized during transcription, identifying details were removed, and pseudonyms were used in reporting the findings. Audio recordings and transcripts were securely stored and accessed only by the research team.

With regard to researcher positionality, the authors include a Full Professor of Special Education and a postdoctoral research fellow in the same disciplinary field. Both have academic and research experience in disability studies and inclusive education. While they had no prior personal relationships with the participants and no direct involvement in the services discussed, their professional backgrounds may have shaped their sensitivity to certain themes related to inclusion, autonomy, and systemic barriers. To ensure analytical rigour and minimize potential bias, reflexive awareness was maintained throughout the research process, and interpretations were continuously examined through peer debriefing and collaborative discussion within the research team. This reflexive stance supported transparency and helped ensure that findings remained grounded in participants' narratives rather than in researchers' assumptions.

## 4 Findings

This section presents a comprehensive overview of the key findings derived from our qualitative interviews with parents of individuals with ID.

### 4.1 Navigating Systemic Challenges in the Transition to Adulthood for Individuals With Intellectual Disabilities

#### 4.1.1 Difficulties in Planning and Implementing a Meaningful “Life Project”

A significant finding of this study centres on the challenges parents encounter in effectively planning and implementing a comprehensive “life project” for their children with ID. While individualized plans are often meticulously drafted, many parents reported that these plans frequently fall short of their intended goals, proving to be inadequate or ill‐suited to their children's evolving needs and aspirations. A core issue was the lack of well‐structured programs, with interventions often dependent not on specialized knowledge and individualized assessment but rather on rigid adherence to a standardized approach. As one parent described:

It wasn't about what my child needed; it was all about fitting him into the standard. If the standard is this, then we do this, period. Sara

This discrepancy between the intended personalized support and the reality of standardized interventions leads to frustration, disillusionment, and a sense of wasted effort, leaving families feeling ill‐prepared for the transition to adulthood.

As one parent stated, encapsulating the widespread sentiment of unmet expectations:

We spent hours working on the ‘life project,’ but it feels like it's just a piece of paper. It doesn't really reflect what my child needs, and no one seems to follow it anyway. Maria

This finding underscores the urgent need for more responsive, flexible, and collaborative approaches to life planning, ensuring that plans are not only tailored to individual needs but also actively implemented and consistently evaluated in partnership with parents.

#### 4.1.2 Challenges in Transition and Employment Pathways

The findings underscore significant impediments in the transition from educational settings to meaningful employment for individuals with ID. Parents consistently reported a discernible absence of clearly defined and structured pathways designed to facilitate this transition, resulting in what many described as a “total void” of viable options in the immediate post‐school phase. This situation is further complicated by the intermittent nature of training programs and internships, which are often disrupted abruptly, thereby exacerbating the instability and insecurity experienced by individuals with ID seeking long‐term employment.

#### 4.1.3 The Need for Enhanced Orientation Services

Parents consistently reported considerable difficulties in navigating the complex landscape of available resources and support services. The current system is often characterized by fragmentation, a lack of transparency, and a scarcity of readily accessible information. This confluence of factors makes it challenging for parents to access essential services, identify appropriate opportunities, and effectively advocate for their children's needs. The reliance on informal networks and word‐of‐mouth underscores the inadequacy of existing formal support structures and highlights the urgent need for streamlined orientation services.

#### 4.1.4 Fostering Autonomy and Developing Essential Skills

While initiatives aimed at fostering autonomy through practical, daily‐life activities have demonstrated positive outcomes, challenges persist in the development of crucial skills. Parents highlighted the difficulties individuals face in mastering concepts such as time management and financial literacy. Moreover, while individuals with ID may exhibit competence in performing specific tasks, their readiness for independent employment without ongoing support remains a concern.

As one parent shared, emphasizing the need for ongoing assistance:

He can do the work, but he needs someone to help him manage his time and stay on task. Luana

These findings underscore the need for comprehensive, tailored interventions that address both practical and cognitive skill development.

#### 4.1.5 The Critical Role of Social Integration and Support Networks

The findings highlight the importance of social integration and the development of meaningful relationships for individuals with ID. Many parents have trouble in establishing and maintaining friendships outside of structured, protected environments. Although organized social activities provide valuable opportunities for interaction, feelings of loneliness and isolation remain a pervasive concern, particularly with regard to long‐term well‐being and future care considerations. As a mother expressed, emphasizing the challenges in facilitating social connections:

She enjoys going to the activities, but it's hard for her to make real friends. She feels lonely sometimes. Marilina

This emphasizes the need for proactive strategies to promote social inclusion and foster supportive networks.

#### 4.1.6 The Overwhelming Burden on Parents and the Call for Systemic Support

Parents consistently voiced concerns regarding the lack of adequate support and guidance, often finding themselves burdened with the responsibility of navigating complex systems and advocating for their children's needs. The absence of a structured support network and clear information regarding legal benefits exacerbates the challenges faced by parents. As one parent stated:

I feel like I'm constantly fighting for him. It's exhausting, and I don't know how much longer I can keep it up. Nicole

Parents emphasized the critical need for systemic support, encompassing financial assistance, psychological counselling, and access to qualified professionals, to alleviate the overwhelming burden on caregivers and ensure the well‐being of both individuals with ID and their parents.

### 4.2 The Lived Emotional Experience of Parents Navigating the Transition to Adulthood for Individuals With Intellectual Disabilities

Beyond the objective systemic challenges, the parental experience is profoundly shaped by a complex interplay of subjective emotions, which both reflect and actively shape their journey. Qualitative analysis of in‐depth interview transcripts revealed key emotional themes that consistently emerged across the parental narratives, including: steadfast resilience in the face of adversity, profound pride in their children's achievements, enduring hope for a brighter future, persistent anxiety about potential challenges, palpable frustration with systemic inadequacies, and an often‐debilitating sense of powerlessness when confronted with seemingly insurmountable obstacles.

#### 4.2.1 Positive Emotional Experiences

##### 4.2.1.1 Resilience

Resilience emerged as a particularly salient and recurrent theme, characterized by parents' unwavering dedication to surmounting obstacles, advocating tirelessly for their children's rights, and proactively seeking innovative solutions to address their unique needs. This was frequently expressed through demonstrable resourcefulness, persistent information‐seeking, and an unyielding willingness to educate others—including educators, service providers, and community members—about their children's specific requirements and challenges. A mother's poignant declaration:

I found myself in the position of having to build everything. Caterina

It encapsulates the proactive, resourceful, and often exhaustive efforts undertaken by many parents to construct the necessary scaffolding of support for their children.

##### 4.2.1.2 Pride

A deep‐seated sense of pride consistently permeated the parental narratives, often manifesting as an overt celebration of their children's unique abilities, personal growth, and incremental accomplishments. Parents routinely emphasized the importance of acknowledging and valuing even seemingly minor achievements as significant milestones in their child's developmental journey. One mother's emphatic declaration, exemplifying the profound satisfaction derived from witnessing her daughter's progress,

Once you teach her, she knows how to do it. Agnese

powerfully illustrates this pervasive sentiment. Furthermore, parents frequently underscored their children's acquisition of essential skills—ranging from basic literacy and numeracy to the mastery of crucial adaptive behaviours instrumental in facilitating greater independence in daily living. The ability of a child to

read and write, to sign [their name], so it's a big thing.

as one mother proclaimed, exemplifies this sense of accomplishment and validation. This unwavering sense of pride serves as a powerful catalyst, motivating parents to continue advocating for their children's needs and striving to unlock their full potential.

##### 4.2.1.3 Enduring Hope and Unwavering Determination

Despite the numerous challenges encountered, parents consistently conveyed a remarkable sense of enduring hope and unwavering determination, fuelled by an unshakeable conviction that a more fulfilling future was attainable for their children. This hope was frequently channelled into proactive efforts to identify and secure personalized educational and vocational opportunities, as well as a dedicated commitment to exploring innovative strategies designed to enhance their children's autonomy, foster their social integration, and improve their overall quality of life. The proactive planning and active pursuit of a comprehensive “After Us” project—aimed at securing a safe, supportive, and enriching living environment for their children in the event of their own incapacitation or passing—exemplifies this enduring hope and resolute determination. As the father declared:

We hope for an autonomous life for our daughter, with a safer and more stable future, which includes a house and adequate support. Carlo

This unyielding hope serves as a powerful source of motivation, enabling parents to persevere in the face of adversity and advocate tirelessly for a more inclusive and equitable future for their children.

#### 4.2.2 Adverse Emotional Experiences

##### 4.2.2.1 Anxiety

Anxiety emerged as a highly prevalent and often debilitating emotion, frequently stemming from deep‐seated concerns regarding their children's academic progress, social integration, overall well‐being, and long‐term prospects for independence. Parents consistently voiced their anxieties regarding the perceived inadequacies of existing support systems, the inherent uncertainties surrounding their children's future self‐sufficiency, and the potential for social isolation and marginalization. As one mother questioned, encapsulating the pervasive uncertainties surrounding post‐school trajectories:

The problem is, what do we do after school is over? It opens up a black hole, because one is not, has not been too prepared. Maria

Moreover, parents frequently expressed anxieties related to their children's vulnerability to exploitation, abuse, and neglect, as well as their own future capacity to provide adequate care and support.

##### 4.2.2.2 Frustration

Parents consistently voiced their frustration with a myriad of aspects of the existing support system, including perceived inadequacies in the level of collaboration among educators, persistent bureaucratic obstacles, the limited availability of suitable and engaging post‐school opportunities, and the often‐fragmented nature of available services. Feelings of disillusionment were frequently exacerbated by a perception that vocational training programs were largely ineffective, that service providers lacked adequate training and resources, and that the system as a whole was often unresponsive to their children's unique needs. As one mother lamented:

The boys are thrown there, let's say without a tutor, without anything. Nicole

Quotes like this highlight the perceived lack of structured support within vocational programs. The difficulties encountered in advocating for appropriate educational accommodations, securing necessary therapies, and navigating complex bureaucratic processes served to further amplify parental frustration and feelings of being overwhelmed.

##### 4.2.2.3 Sense of Powerlessness

Many parents openly expressed a deep‐seated sense of powerlessness in the face of systemic barriers, institutional inertia, and persistent societal prejudices. This feeling of disempowerment was often compounded by legitimate concerns about their own aging and the potential erosion of their capacity to provide adequate care and advocacy for their children in the years to come. As one parent expressed, highlighting their fears about the future.

We parents will not be forever able to understand, to want… We are getting tired too, the energy is not always that of thirty years. Camilla

The seemingly endless cycle of fighting for their children's rights, navigating complex bureaucratic processes, and battling against discriminatory attitudes frequently left parents feeling exhausted, demoralized, and utterly overwhelmed.

## 5 Discussion

This study contributes to the literature on transition to adulthood for individuals with intellectual disabilities (ID) in several original ways.

First, it offers one of the earliest empirical explorations of parental experiences in the context of Italy's recent policy reform introducing the “Life Project” (LP) under Legislative Decree 62/2024. While existing scholarship has extensively documented systemic fragmentation and barriers to employment (e.g., Floyd et al. 2009; Lindstrom et al. 2013), this research situates such challenges within an evolving legislative framework, revealing a critical implementation gap between policy aspirations and parents' lived realities. In doing so, it provides timely evidence to inform the operationalization of the LP. Parents' narratives largely reflect experiences developed prior to the formal implementation of the LP, highlighting pre‐existing systemic gaps. As such, the findings should be interpreted as capturing a ‘pre‐implementation landscape’, against which the LP can be critically evaluated. Rather than reflecting direct experiences of the LP in practice, parents' accounts reveal the structural and relational conditions into which the LP is being introduced. In this sense, the findings illuminate the gaps that the LP is intended to address, while also raising critical questions about its implementation feasibility.

Second, the study advances current knowledge by conceptualizing the transition to adulthood not merely as a service coordination issue but as an emotionally mediated systemic process. Rather than treating parental resilience, anxiety, pride, and powerlessness as secondary outcomes, the findings demonstrate how these emotional experiences are structurally produced through institutional interactions and uncertainty about future pathways. This perspective reframes parental emotions as indicators of systemic functionality, offering a more integrated socio‐emotional lens on transition processes.

Third, the research introduces the notion of *anticipatory discontinuity* to describe parents' perception of a “void” after compulsory schooling. This contribution extends the literature by emphasizing the temporal dimension of transition: the shift from structured, predictable educational environments to an unstructured and opaque adult service landscape. The findings suggest that transition planning must address not only vocational outcomes but also continuity, identity development, and long‐term life design.

Finally, by interweaving employment access, service navigation, emotional sustainability, and community inclusion within a single analytic framework, the study proposes a genuinely holistic, family‐centred model of support. This integrated approach moves beyond sector‐specific recommendations and underscores the need for coordinated, co‐constructed planning processes that position parents as active partners rather than peripheral stakeholders.

### 5.1 Strengths and Limitations

This study possesses several strengths. The use of qualitative interviews allowed for a rich and in‐depth exploration of parental experiences, capturing nuances and complexities that may not be accessible through quantitative methods alone. By integrating both systemic challenges and parental emotions, it offers a holistic perspective on navigating the transition to adulthood for individuals with ID. The research also highlights the potential of the LP framework (Legislative Decree 2024) and identifies key systemic barriers, providing valuable insights for policy and practice improvements.

However, it is important to acknowledge certain limitations. As is characteristic of qualitative research, the aim of this study was not to achieve generalizability but to provide deep, contextual insights. Therefore, the findings are not generalizable to the broader population but may be transferable to similar contexts.

### 5.2 Implications and Future Directions

The findings of this study could have concrete implications for policy and practice, demanding targeted interventions to improve the transition to adulthood for individuals with ID and support their parents. Given the reported lack of structured pathways to employment, there is an urgent need to develop and implement targeted vocational training programs tailored to the individual skills and interests of individuals with ID. These programs should prioritize supported internships and on‐the‐job training, focusing on transferable skills and fostering employer partnerships. The difficulties parents face navigating the complex service system highlight the necessity of establishing centralized information and navigation hubs providing comprehensive, accessible information about available resources, eligibility, and application processes, alongside personalized support. Recognizing the emotional toll on caregivers, integrated family support services are crucial, offering access to respite care, counselling, peer support groups, and training on advocacy, legal rights, and financial planning. To combat social isolation, community inclusion initiatives are essential, fostering opportunities for individuals with ID to participate in social, recreational, and civic activities with their peers, while raising awareness and promoting inclusive attitudes. Finally, further research is needed to rigorously evaluate and refine the LP framework, identifying best practices for implementation and ensuring that plans are truly individualized, responsive, and actively implemented in partnership with parents. Future research should also explore the long‐term impacts of these interventions and prioritize the voices of individuals with ID in the development of future policies and programs.

## 6 Conclusion

Navigating the transition to adulthood for individuals with ID is marked by systemic challenges that significantly impact the parental experience. These challenges are interwoven with a complex emotional landscape, underscoring the critical need for comprehensive support systems that address both practical and emotional needs to foster family resilience and improve outcomes. Understanding the emotional experiences of parents of individuals with ID is paramount for developing effective support strategies. The LP, as a personalized and participatory framework, holds significant potential for promoting autonomy, inclusion, and improved quality of life for individuals with ID. Addressing existing gaps in support services and empowering parents are crucial steps towards realizing the full potential of the LP and fostering a more inclusive and equitable society. By acknowledging and addressing the multifaceted challenges highlighted in this study, we can work towards creating a future where individuals with ID and their parents have the opportunity to thrive.

## Funding

The authors have nothing to report.

## Ethics Statement

This study has been conducted in accordance with the ethical standards outlined in the ethical code for research in Pedagogy and it received ethical approval from the Ethical Committee of the University of Trieste (Extract from report n. 3 of March 27th, 2024).

## Conflicts of Interest

The authors declare no conflicts of interest.

## Data Availability

The data that support the findings of this study are available from the corresponding author upon reasonable request.
